# Type I interferon pathway activation across the antiphospholipid syndrome spectrum: associations with disease subsets and systemic antiphospholipid syndrome presentation

**DOI:** 10.3389/fimmu.2024.1351446

**Published:** 2024-03-14

**Authors:** Irene Cecchi, Massimo Radin, Alice Barinotti, Silvia Grazietta Foddai, Elisa Menegatti, Dario Roccatello, Ana Suárez, Savino Sciascia, Javier Rodríguez-Carrio

**Affiliations:** ^1^ University Center of Excellence on Nephrologic, Rheumatologic and Rare Diseases (ERK-Net, ERN-Reconnect and RITA-ERN Member) with Nephrology and Dialysis Unit and Center of Immuno-Rheumatology and Rare Diseases (CMID), Coordinating Center of the Interregional Network for Rare Diseases of Piedmont and Aosta Valley, San Giovanni Bosco Hub Hospital, Turin, Italy; ^2^ Department of Clinical and Biological Sciences, University of Turin, Turin, Italy; ^3^ Area of Immunology, Department of Functional Biology, Faculty of Medicine, University of Oviedo, Oviedo, Spain; ^4^ Area of Metabolism, Instituto de Investigación Sanitaria del Principado de Asturias (ISPA), Oviedo, Spain

**Keywords:** antiphospholipid syndrome, systemic lupus erythematosus, interferon, interferon signature, antiphospholipid antibodies

## Abstract

**Introduction:**

While the type I interferon (IFN-I) pathway is crucial in autoimmunity, its role in antiphospholipid antibody (aPL)-positive subjects, including aPL carriers and antiphospholipid syndrome (APS) patients, is poorly understood. This study aims at characterizing IFN-I pathway activation within the spectrum of aPL-positive subsets.

**Methods:**

A total of 112 patients [29 aPL carriers, 31 primary APS (PAPS), 25 secondary APS (SAPS), 27 systemic lupus erythematosus (SLE) patients without aPL, and 44 healthy controls (HCs)] were recruited. IFI6, IFI44, IFI44L, MX1, IFI27, OAS1, and RSAD2 gene expression was evaluated in whole blood, and a composite index (IFN score) was calculated.

**Results:**

An overall activation of the IFN-I pathway was observed across the entire APS spectrum, with differences among genes based on the specific disease subset. The composite score revealed quantitative differences across subsets, being elevated in aPL carriers and PAPS patients compared to HCs (both *p* < 0.050) and increasing in SAPS (*p* < 0.010) and SLE patients (*p* < 0.001). An unsupervised cluster analysis identified three clusters, and correspondence analyses revealed differences in clusters usage across APS subsets (*p* < 0.001). A network analysis revealed different patterns characterizing different subsets. The associations between IFN-I pathway activation and clinical outcomes differed across APS subsets. Although no differences in gene expression were observed in systemic APS, the network analyses revealed specific gene–gene patterns, and a distinct distribution of the clusters previously identified was noted (*p* = 0.002).

**Conclusion:**

IFN-I pathway activation is a common hallmark among aPL-positive individuals. Qualitative and quantitative differences across the APS spectrum can be identified, leading to the identification of distinct IFN-I signatures with different clinical values beyond traditional categorization.

## 1 Introduction

The clinical definition of antiphospholipid syndrome (APS) relies on the finding that individuals persistently positive for antiphospholipid antibodies (aPL), including lupus anticoagulant (LA), anti-β2 glycoprotein I (aβ2GPI), and anti-cardiolipin (aCL) antibodies. APS patients are at a higher risk than the general population to develop arterial and venous thrombotic events, especially at a young age (1, 2). Moreover, women with APS can experience recurrent pregnancy losses along with several fetal and maternal complications, such as preterm delivery, intrauterine growth restriction, and preeclampsia (1). Indeed thrombotic and obstetric phenotypes, which can coexist within the same subject, constitute a distinct clinical entity known as “primary APS” (PAPS). The association between APS and other autoimmune conditions, such as systemic lupus erythematosus (SLE), which further complicates the management of these patients, is commonly called “secondary APS” (SAPS). Although this nosological approach is useful to categorize individuals into discrete disease subgroups based on a number of shared clinical and serological features, compelling evidence suggest that it does not encompass the entire clinical spectrum of the disease, thus leaving a non-negligible part of patients uncovered and/or underdiagnosed. In fact, over the years, a deeper understanding of the syndrome has led to the identification of a wide range of overlapping additional clinical manifestations as well as novel potential biomarkers, mirroring the complexity of APS pathophysiology, which seems far from being fully elucidated (3).

Several attempts have been made to overcome the conventional classification of the syndrome, both from a clinical and biological standpoint, with the aim of profiling rather than categorizing patients. Among them, two recent publications (4, 5) have described the existence of a bridging condition, often encountered in clinical practice, between pure thrombotic APS and SLE, which was termed “systemic APS”. This subgroup of patients is mainly characterized by a persistent aPL positivity, with or without APS, and additional non-aPL-related clinical and laboratory manifestations such as cytopenia and anti-nuclear antibodies (ANA) positivity. Further research is needed to precisely define this condition, which, however, highlights a continuum into the APS spectrum and confirms the heterogeneous phenotype among aPL-positive patients beyond thrombosis and pregnancy complications. The correct identification of these aPL-positive individuals, with or without previous thrombotic events, who do not fulfil the diagnostic criteria for a defined connective tissue disorder despite presenting a tendency toward a more systemic involvement might lead to alternative therapeutic strategies, such as the use of immunomodulant agents, monitoring, and prognosis.pt?>

From a molecular perspective, type I interferons (IFN-I) have been associated with the breakdown of tolerance and perpetuation of autoimmune responses (6). Although extensive data has supported their involvement in a number of systemic autoimmune conditions, a recent systematic review has revealed that APS has received limited attention (7, 8). Emerging data have suggested the importance of IFN-I in the pathogenesis of APS (9), especially in the established stage. However, whether IFN-I pathway activation underlies the earliest stages of the disease and its clinical significance have not been explored yet. Evidences from other conditions, such as SLE (10–12), have confirmed IFN-I pathway’s promise to improve disease monitoring and patient stratification as well as to drive disease profiling approaches. Nevertheless, methodological challenges and the low number of studies available pose additional challenges to understand the potential use of IFN-I pathway activation in APS (7).

Taken together, we hypothesize that IFN-I pathway activation may help in the profiling of the APS spectrum and gain insight into their clinical classification. The overarching aim of this study was to provide new insights into IFN-I pathway activation in APS in relation to its clinical relevance. The specific aims were (i) to assess the IFN-I pathway activation in a cohort of aPL-positive individuals, including patients affected by well-described nosological entities such as PAPS and SAPS, as well as SLE patients, (ii) to evaluate the associations between the degree of activation and the structure of the IFN-I pathway activation with clinical outcomes across the APS spectrum, and (iii) to characterize the IFN-I pathway activation in the systemic APS subset.

## 2 Materials and methods

### 2.1 Ethical approval

The study protocol was performed in compliance with the Declaration of Helsinki and approved by the Institutional Review Boards from the University of Turin and the University of Oviedo (reference CEImPA 2021.126). All participants gave written informed consent prior to enrollment.

### 2.2 Study participants

This cross-sectional study included consecutive patients attending the San Giovanni Bosco Hospital in Turin (Italy) from January 2019 to December 2022. We enrolled patients who met one of the following inclusion criteria:

1) tested persistently positive for at least one criteria aPL (1), in the absence of clinical manifestations of APS (“aPL carriers”);
2) diagnosis of PAPS defined as per Sydney criteria (1);
3) diagnosis of SAPS defined as per Sydney criteria (1);
4) diagnosis of SLE following the 2019 EULAR/ACR classification criteria (13) upon testing persistently negative for criteria aPL as well as for anti-phosphatidylserine/prothrombin (aPS/PT) antibodies (IgG and/or IgM isotypes).

For the purpose of the study, we also included age- and sex-matched subjects as healthy controls (HCs). A systemic APS subset was defined according to the literature as follows (5): 1) persistent aPL positivity with or without clinical manifestations of APS (1), 2) ANA positivity, confirmed over time, tested with immunofluorescence on Hep-2 cells at a titer ≥1:80, 3) at least one additional clinical manifestation (including cytopenia as a whole, hemolytic anemia, leukopenia and thrombocytopenia, hypocomplementemia, arthritis, serositis, Raynaud’s phenomenon, photosensitivity, *livedo reticularis*, and neuropsychiatric and mucocutaneous manifestations related to the presence of an autoimmune condition), and 4) not fulfilling the classification criteria for a defined connective tissue disorder.

Demographic, clinical, and laboratory characteristics were collected at the time of enrollment. The patients and controls were tested for complete aPL profile, including criteria aPL (LA, aCL IgG/IgM, aβ2GPI IgG/IgM), and aPS/PT IgG/IgM antibodies according to validated practice. The aCL, aβ2GPI, and aPS/PT were semi-quantitatively assayed using a validated commercial ELISA kit by Inova Diagnostics, Inc. (San Diego, CA, USA). Cut-off values of positivity were defined following the manufacturer’s instructions. Plasma samples were tested for the presence of LA according to the recommended criteria from the International Society on Thrombosis and Haemostasis Subcommittee on Lupus Anticoagulant/Phospholipid-Dependent Antibodies (14).

The cumulative Global Antiphospholipid Syndrome Score (GAPSS) was calculated for each patient as previously reported by adding together all points corresponding to the score risk factors (15): specifically, five points for aCL (IgG/IgM), four points for LA and aβ2GPI (IgG/IgM), three points for aPS/PT (IgG/IgM) and hyperlipidemia, and one point for arterial hypertension.

### 2.3 RNA isolation and PCR assays

Whole-blood samples were processed immediately after extraction by using RNA Stabilization Reagent for Blood/Bone Marrow (Roche, Germany) for stabilization, according to the protocol provided by the manufacturer and stored at -20°C. The samples were then thawed at room temperature in batches and mRNA was isolated by using the mRNA Isolation Kit for Blood/Bone Marrow (Roche), following the manufacturer’s instructions. Reverse transcription was performed using the Transcriptor First Strand cDNA Synthesis Kit (Roche).

IFN-stimulated genes (ISGs) expression was evaluated as previously described (16). In brief, gene expression was assessed with TaqMan pre-designed assays for the following genes: IFI6 (interferon alpha-inducible protein 6, ref. Hs00242571_m1), IFI44 (interferon-induced protein 44, ref. Hs00197427_m1), IFI44L (interferon-induced protein 44 like, ref. Hs00915292_m1), MX1 (MX dynamin like GTPase 1, ref. Hs00895608_m1), IFI27 (interferon alpha-inducible protein 27, ref. Hs01086373_g1), OAS1 (2′–5′-oligoadenylate synthetase 1, ref. Hs00973635_m1), and RSAD2 (radical S-adenosyl methionine domain containing 2, ref. Hs00369813_m1). These candidate genes were selected based on previous evidence supporting their IFN-I dependency and being reported in APS and SLE studies (8). Real-time quantitative PCR reactions were carried out in an ABI Prism HT7900 (Applied Biosystems, Germany). All samples were assayed in triplicate. Ct values were evaluated with the software SDS 2.3^®^, and expression levels were evaluated by using the 2^-ΔΔCt^ method, using the GAPDH gene expression as a housekeeping (16).

### 2.4 Statistical analysis

Variables were summarized as median (interquartile range) or *n* (%) as appropriate. Z-scores were calculated for each ISG. Differences among groups were assessed by using Mann–Whitney *U*, Kruskal–Wallis (with Dunn–Bonferroni correction for multiple comparisons), or chi-square tests. Correlations were analyzed by using Spearman ranks test. Principal component analysis (correlation method) was used to evaluate collinearity among individual ISGs. A composite index for IFN-I pathway activation (ISG expression score, IFN score) was calculated by averaging all ISGs per individual. Network analyses were generated to analyze the correlations among ISGs across different subsets. Centrality measures (betweenness, closeness, strength, and expected influence) were computed. Unsupervised cluster analysis was performed based on squared euclidean distances and Ward’s minimum variance method. Correspondence analyses were used to explore the simultaneous associations among categorical variables (clusters vs. subsets). A *p*-value <0.050 was considered as statistically significant. Statistical analyses were performed in SPSS 27.0, R 4.1.3, and GraphPad Prism 8.4 for Windows.

## 3 Results

### 3.1 Patients’ characteristics

A total of 112 patients, including 29 aPL carriers, 31 PAPS, 25 SAPS, and 27 SLE patients without aPL positivity, were recruited. The mean age at inclusion was 48.5 years (SD ± 13.5 years), with an expected female predominance (75%). In addition, a total of 44 HCs were included in the analysis. The complete demographic, clinical, and laboratory characteristics at the time of inclusion in the study and at sample collection are displayed in **Table 1**.

**Table 1:** Description of the study participants.

	HCs	aPL carriers	PAPS	SAPS	SLE
Total number of patients	44	29	31	25	27
Demographic features					
Age, years, mean ( ± SD)	50 (11)	46.5 (13)	54 (13)	49 (12)	41 (10)
Sex, females, *n* (%)	39 (89)	22 (75)	25 (81)	14 (56)	23 (85)
Ethnicity, Caucasians, *n* (%)	44 (100)	29 (100)	30 (96)	23 (92)	27 (100)
Clinical features					
Disease duration, years, mean ( ± SD)	–	–	10 (6)	18 (11)	13 (9)
Thrombosis (arterial and/or venous), *n* (%)	0	0	28 (90)	25 (100)	3 (11)
Thrombotic recurrences, *n* (%)	0	0	6 (19)	5 (20)	1 (3)
Obstetric complications (APS criteria^a^), *n* (%)	0	0	5 (16)	1 (4)	0
Serologic features					
aPL-positive, *n* (%)	0	29 (100)	31 (100)	25 (100)	0
aCL-positive (IgG/IgM), *n* (%)	0	14 (48)	25 (81)	14 (56)	0
aβ2GPI-positive (IgG/IgM), *n* (%)	0	10 (34)	21 (68)	12 (48)	0
LA-positive, *n* (%)	0	20 (69)	25 (81)	19 (76)	0
aPS/PT-positive (IgG/IgM), *n* (%)	0	13 (45)	15 (48)	16 (64)	0
Hypocomplementemia (C3 and/or C4 fractions), n (%)	–	11 (38)	5 (16)	12 (48)	18 (67)
ANA-positive, *n* (%)	–	16 (55)	17 (31)	25 (100)	27 (100)
Anti-dsDNA-positive, *n* (%)	–	1 (3)	0	16 (64)	22 (81)
ENA-positive, *n* (%)	–	7 (24)	1 (3)	8 (32)	14 (52)
Traditional cardiovascular risk factors and GAPSS^b^					
Arterial hypertension, *n* (%)	9 (20)	9 (31)	17 (31)	10 (32)	8 (30)
Dyslipidemia, *n* (%)	8 (18)	4 (14)	13 (42)	10 (32)	2 (7)
Diabetes mellitus, *n* (%)	1 (2)	0	6 (19)	2 (8)	0
Smoking (ongoing), *n* (%)	5 (11)	3 (10)	7 (22.5)	9 (36)	4 (15)
GAPSS^b^, value, mean ( ± SD)	–	9 (5)	9 (5)	12 (5)	–
Treatment (at the time of sample collection)					
Prednisone or equivalent ≤5 mg/day, *n* (%)	0	9 (31)	6 (19)	12 (48)	17 (63)
Prednisone or equivalent >5 mg/day, *n* (%)	0	1 (3)	0	2 (5)	4 (15)
HCQ (200–400 mg/day), *n* (%)	0	9 (5)	8 (25)	16 (64)	12 (44)
LDA (100 mg/day), *n* (%)	0	14 (48)	20 (64)	14 (56)	4 (15)
Vitamin K antagonists, *n* (%)	0	3 (10)	20 (64)	11 (44)	1 (4)
DOACs, *n* (%)	0	0	4 (13)	4 (16)	0
Other immunosuppressive treatment, *n* (%)	0	2 (7)	2 (6)	10 (32)	10 (37)

HCs, healthy controls; aPL, antiphospholipid antibodies; PAPS, primary antiphospholipid syndrome; SAPS, secondary antiphospholipid syndrome; SLE, systemic lupus erythematosus; aCL, anti-cardiolipin antibodies; aβ2GPI, anti-β2-glycoprotein I antibodies; LA, lupus anticoagulant; aPS/PT, anti-phosphatidylserine/prothrombin antibodies; ANA, anti-nuclear antibodies; anti-dsDNA, anti-double stranded DNA antibodies; ENA, extractable nuclear antigens; GAPSS, Global Antiphospholipid Syndrome Score; HCQ, hydroxychloroquine; LDA, low-dose aspirin; DOACs, direct oral anticoagulants.

a Miyakis S, et al. J Thromb Haemost, 2006.

b Sciascia S, et al. Rheumatology (Oxford), 2013.

Demographic, clinical, and serological features of the study participants.

### 3.2 IFN-I pathway activation across the APS spectrum

**Figure 1:**
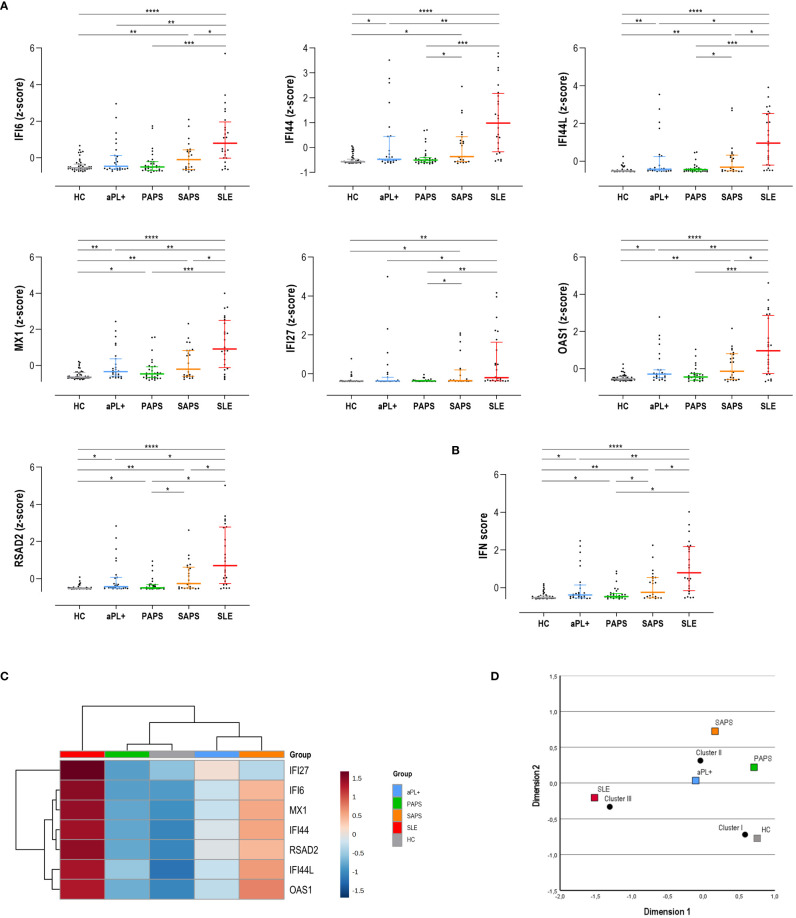
Interferon (IFN) pathway activation across the antiphospholipid syndrome (APS) spectrum. The IFN pathway activation measured as individual IRG **(A)** or as a composite score **(B)** was compared among APS subsets. Results are shown as scatter plots, where lines represent the 25th, 50th (median), and 75^th^ percentiles, and each dot represents one individual. Differences were evaluated by using Kruskal–Wallis test with Dunn–Bonferroni test for multiple comparisons. The *p*-values correspond to those obtained in the multiple-comparisons tests and are indicated as follows: *p < 0.050, **p < 0.010, ***p < 0.001, and ****p < 0.0001. **(C)** A group-averaged (columns) heat map based on the expression of the IRG (rows). The top bar indicates the APS subsets, as per the group legend (right). Tile colors are based on gene expression levels, with red and blue indicating low or high levels, respectively, as per the column legend. The vertical and horizontal dendrograms show the clustering patterns among disease subsets and IRG, respectively. **(D)** Correspondence analysis showing the associations between disease subsets (colored squares) and the three clusters identified (black dots). The axes represent the dimensions derived from the analysis.

The analysis of ISGs expression, either individually (**Figure 1A**) or as a composite score (IFN score) (**Figure 1B**), revealed a significant IFN-I pathway activation across the APS spectrum, although differences were noted among genes and subsets. Interestingly, the expression of some ISGs, such as IFI44, IFI44L, MX1, OAS1, and RSAD2, was increased already in the aPL carriers’ subset compared to HCs (**Figure 1A**). It should be noted that this group exhibited a significant heterogeneity. On the contrary, other ISGs were found to be increased only in SLE or SAPS subsets, such as IFI6 or IFI27. Although no changes were observed between aPL carriers and PAPS subsets in any of the genes analyzed, IFI44 and OAS1 were found to be elevated in aPL carriers compared to HCs, whereas the same cannot be applied to their PAPS counterparts. Similarly, certain ISGs (IFI44, IFI44L, IFI27, and RSAD2) showed differences between PAPS and SAPS subsets. Finally, a significant number of ISGs (IFI6, IFI44L, MX1, OAS1, and RSAD2) exhibited differences between SAPS and SLE patients. As expected, SLE patients exhibited the highest and more consistent IFN-I pathway activation.

As expected, all ISGs showed a high degree of correlation. This was confirmed by means of a PCA (matrix determinant: *p* = 2.16·10^-6^ and KMO = 0.915, *p* < 10^-10^). All ISGs showed communalities higher than 0.9, with the exception of IFI27 (0.536). However, only one component was extracted, accounting for 85.9% of the total variance and with all ISGs having loadings >0.9 except for IFI27 (0.756). Then, after confirming the high collinearity of all ISGs analyzed, the IFN score was computed. The composite score revealed quantitative differences across APS subsets, being elevated in aPL carriers and PAPS subsets compared to HCs (both *p* < 0.050) and increasing in SAPS (*p* < 0.010) and SLE (*p* < 0.001) (**Figure 1B**).

An unsupervised cluster analysis built with the individual ISGs revealed the identification of three clusters (referred to as clusters I to III) (**Figure 1C**). Interestingly, the aPL carriers group clustered closer to SAPS, whereas PAPS did with HCs. SLE patients showed the highest differences with the rest of the groups entered in the analysis. Importantly, the correspondence analyses demonstrated that cluster usage differed across APS subsets (*p* < 0.001), thus correlating with different clinical status (**Figure 1D**). aPL carriers localized closer again to cluster II, although in a less divergent position (closer to the graph center) compared to both PAPS and SAPS.

**Figure 2:**
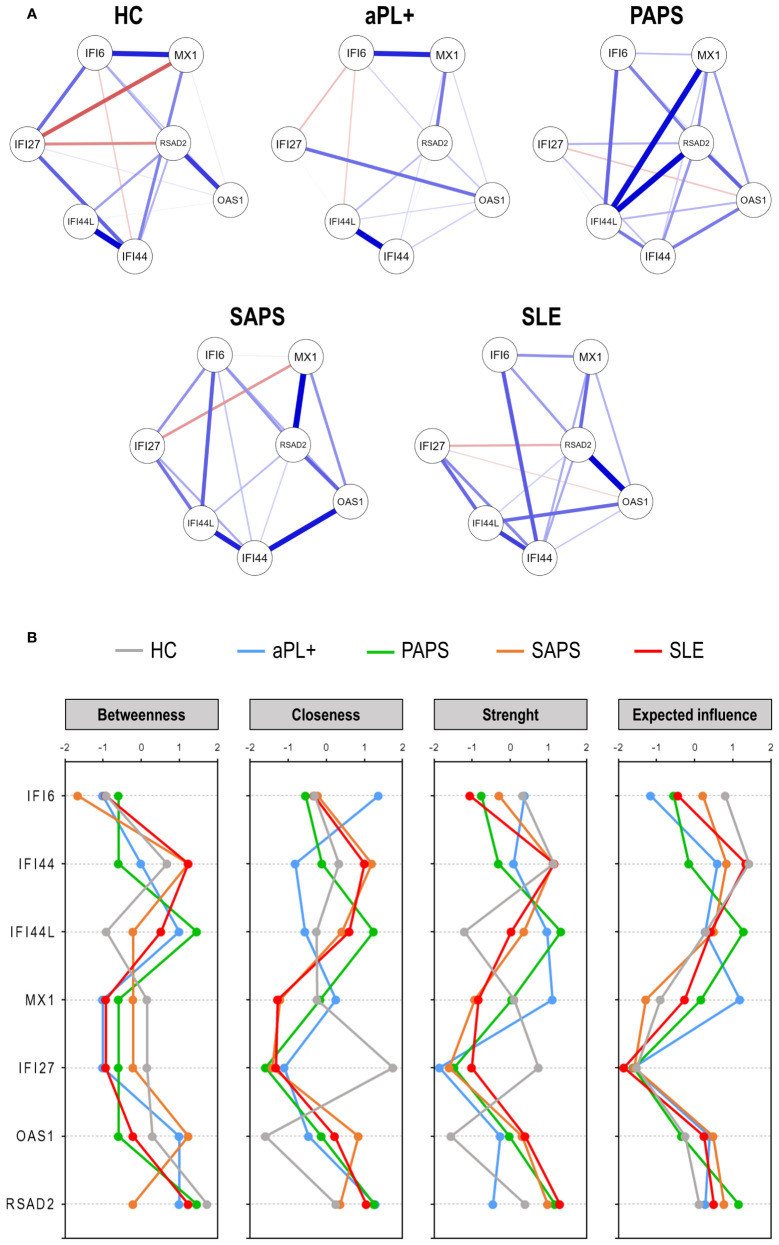
Network analyses of interferon pathway activation patterns across the antiphospholipid syndrome (APS) spectrum. **(A)** Network analyses depicted based on the gene–gene correlations among APS subsets. Each node corresponds to a single gene, and the lines between nodes illustrate the strength (width) and type (blue: positive, red: negative) of the correlations between each pair of genes. **(B)** Centrality measures (betweenness, closeness, strength, and expected influence) of the IRG network analyses. IFN-stimulated genes are indicated in the vertical axes, and centrality measures are represented in the horizontal axes for each study group [lines colored as per plot legend (top)].

Finally, network graphs were generated to evaluate the gene–gene interactions (**Figure 2A**). These analyses revealed that different pictures hallmarked the different subsets. HCs exhibited a uniform network, also showing negative correlations. On the contrary, APS subsets exhibited more heterogeneous networks, mostly composed of positive correlations. Interestingly, certain associations were observed to be differentially enriched across subsets (IFI44L and RSAD2 in PAPS, IFI44 and OAS1 in SAPS, or RSAD2 and OAS1 in SLE). The sparsity and degree of the networks increased from aPL carriers (fuzzy pattern) to SLE (strong and high degree network), as the number, strength, and edge locations did. These findings were supported by centrality measures, with higher differences across groups being found for IFI44, IFI44L, MX1, and OAS1 (**Figure 2B**). Centrality measures confirmed similar patterns for SAPS and SLE, especially for closeness and strength, whereas a highly heterogenous profile was observed for aPL carriers. PAPS lie in between these groups.

Taken together, all these results support an early and progressive IFN-I pathway activation across the APS spectrum, where quantitative and qualitative differences were observed. The expression of ISGs delineated certain clinically relevant clusters which paralleled nosological status.

### 3.3 IFN-I pathway activation and clinical features across APS subsets

Next, the associations between ISGs and IFN score with several clinical features were evaluated across APS subsets.

Thrombosis occurrence (arterial or venous) was unrelated to IFN-I pathway activation, either measured by individual ISGs expression or as a composite score (**Supplementary Table S1**). However, the presence and extent of recurrence of thrombosis were positively associated with the expression of IFI44, OAS1, and RSAD2 as well as with the IFN score in patients with SAPS (**Supplementary Table S1**). No effect was noted in the rest of the groups. Moreover, the IFN score was unrelated to GAPSS across the APS spectrum (aPL: *r* = 0.224, *p* = 0.261; PAPS: *r* = 0.028, *p* = 0.880; SAPS: *r* = -0.026, *p* = 0.907; and SLE: *r* = -0.276, *p* = 0.214). Similarly, no associations with total white blood cell count were found (aPL: *r* = 0.052, *p* = 0.839; PAPS: *r* = 0.048, *p* = 0.818; SAPS: *r* = -0.299, *p* = 0.229; and SLE: *r* = 0.008, *p* = 0.974). Equivalent findings were observed when ISGs were analyzed individually (data now shown).

The presence of criteria aPL (LA, aCL, and aβ2GPI) was not found to be associated with the IFN-I pathway activation in any of the APS subsets (**Supplementary Table S2**), but when computed in terms of the aPL profile, triple aPL positivity was associated with enhanced IFN-I pathway activation only in aPL carriers (IFN score: *p* = 0.050), although differences were found among ISGs (**Supplementary Table S3**). However, no associations were observed in the rest of the subsets (**Supplementary Table S3**). No effect was observed for double positivity across disease subsets (**Supplementary Table S3**). The correlation analyses between the number of criteria aPL and IFN-I pathway activation revealed no dose-dependent effect on the latter (**Supplementary Table S3**). Furthermore, ANA positivity was found to be associated with IFN-I pathway activation in aPL carriers but showed no impact on the rest of the subsets (**Supplementary Table S2**). Additionally, the levels of aPS/PT IgG antibodies strongly correlated with IFN-I pathway activation in aPL carriers and, to a lesser extent, in PAPS patients (**Supplementary Table S2**), with no effect in SAPS and SLE groups. Similar findings were retrieved with the IgM isotype.

Finally, the effect of medications on IFN-I pathway activation was assessed. It is worth noting that no effects of treatments were registered across the APS spectrum (**Supplementary Table S4**).

In conclusion, these findings suggest that although certain associations between IFN-I pathway activation and clinical features may be found, these are restricted to specific APS subsets, thereby pointing to a certain heterogeneity in IFN-I pathway activation that may influence clinical value.

### 3.4 Characterizing systemic APS through IFN-I pathway activation

Next, IFN-I pathway activation was evaluated in systemic APS. A total of nine patients from our cohort were considered as having systemic APS and were compared with those not having systemic APS (**Table 2**).

**Table 2:** Characteristics of systemic antiphospholipid syndrome (APS) cohort.

	Systemic APS		*p*-value
	No	Yes	
Total number of patients	48	9	
Demographic features			
Age, years, mean ( ± SD)	51.5 (14)	53 (14)	n.s.
Sex, females, *n* (%)	25 (52)	5 (55)	n.s.
Ethnicity, Caucasians, *n* (%)	47 (98)	9 (100)	n.s.
Clinical features			
Disease duration, years, mean ( ± SD)	10 (6)	13 (8)	n.s.
Thrombosis (arterial and/or venous), *n* (%)	20 (42)	7 (78)	n.s.
Thrombotic recurrences, *n* (%)	3 (6)	2 (22)	n.s.
Obstetric complications (APS criteria^a^), *n* (%)	4 (8)	1 (11)	n.s.
Arthritis, *n* (%)	0	2 (22)	–
Serositis, *n* (%)	0	0	–
Mucocutaneous manifestations, *n* (%)	1 (2)	2 (22)	n.s.
Raynaud’s phenomenon, *n* (%)	0	1 (11)	–
Photosensitivity, *n* (%)	1 (2)	1 (11)	n.s.
*Livedo reticularis*, *n* (%)	0	1 (11)	–
Neuropsychiatric manifestations, *n* (%)	1 (2)	0	–
Serological features			
aPL-positive, *n* (%)	48 (100)	12 (100)	n.s.
aCL-positive (IgG/IgM), *n* (%)	22 (46)	7 (78)	n.s.
aβ2GPI-positive (IgG/IgM), *n* (%)	17 (35)	7 (78)	0.040
LA-positive, *n* (%)	25 (52)	6 (67)	n.s.
aPS/PT positive (IgG/IgM), *n* (%)	13 (27)	4 (45)	n.s.
Hypocomplementemia (C3 and/or C4 fractions), *n* (%)	2 (4)	4 (45)	0.002
Autoimmune hemolysis, *n* (%)	0	3 (33)	–
Leukopenia, *n* (%)	0	3 (33)	–
Thrombocytopenia, *n* (%)	2 (4)	7 (78)	<0.001
ANA-positive, *n* (%)	11 (23)	9 (100)	–
Anti-dsDNA-positive, *n* (%)	0	0	–
ENA positive^b^, *n* (%)	2 (4)	2 (22)	n.s.
Traditional cardiovascular risk factors and GAPSS^c^			
Arterial hypertension, *n* (%)	17 (35)	4 (45)	n.s.
Dyslipidemia, *n* (%)	10 (21)	6 (67)	0.020
Diabetes mellitus, *n* (%)	3 (6)	2 (22)	n.s.
Smoking (ongoing), *n* (%)	9 (19)	0	–
GAPSS, value, mean ( ± SD)^c^	11 (5)	12 (4)	n.s.
Treatment (at the time of sample collection)			
Prednisone or equivalent ≤5 mg/day, *n* (%)	4 (8)	3 (33)	n.s.
Prednisone or equivalent >5 mg/day, *n* (%)	0	1 (11)	–
HCQ (200–400 mg/day), *n* (%)	8 (17)	2 (22)	n.s.
LDA (100 mg/day), *n* (%)	16 (33)	7 (78)	0.030
Vitamin K antagonists, *n* (%)	13 (27)	7 (78)	0.010
DOACs, *n* (%)	3 (6)	0	n.s.
Other immunosuppressive treatment, *n* (%)	3 (6)	2 (22)	n.s.

APS, antiphospholipid syndrome; aCL, anti-cardiolipin antibodies; aβ2GPI, anti-β2-glycoprotein I antibodies; LA, lupus anticoagulant; aPS/PT, anti-phosphatidylserine/prothrombin antibodies; ANA, anti-nuclear antibodies; anti-dsDNA, anti-double stranded DNA antibodies; ENA, extractable nuclear antigens; GAPSS, Global Antiphospholipid Syndrome Score; HCQ, hydroxychloroquine; LDA, low-dose aspirin; DOACs, direct oral anticoagulants; n.s., not significant.

a Miyakis S, et al. J Thromb Haemost, 2006.

b We excluded patients who tested positive for anti-Smith antibodies based on their high specificity for systemic lupus erythematosus diagnosis.

c Sciascia S, et al. Rheumatology (Oxford), 2013.

The patients were stratified according to their systemic APS status, and their demographic, clinical, and serological features were compared. The variables were summarized as mean ( ± SD) or n (%), unless otherwise stated. Differences were evaluated by using Mann–Whitney U or chi-square tests (using Yates correction) as appropriate.

**Figure 3:**
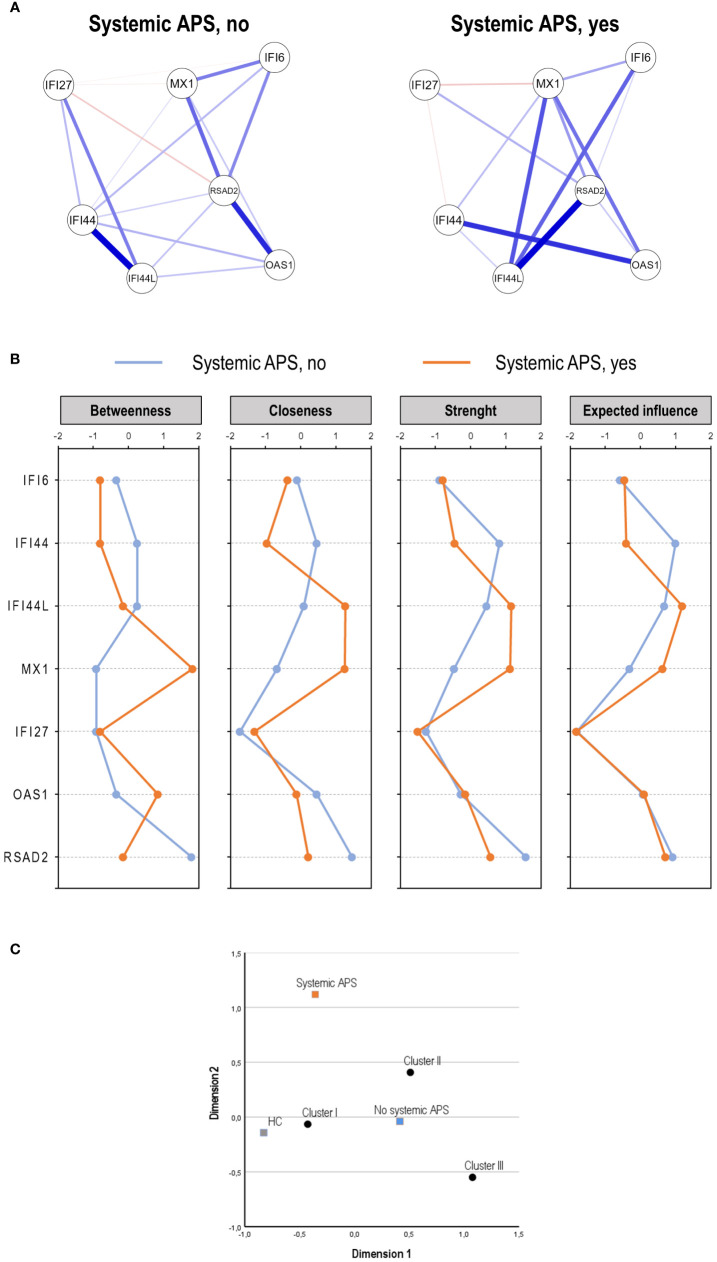
Analysis of the interferon (IFN) pathway activation in the systemic antiphospholipid syndrome (APS) subset. The IFN pathway activation according to systemic APS status (no vs. yes) was evaluated by network analysis **(A)**, centrality measures **(B)**, and correspondence analysis **(C)**.

No differences in ISGs expression levels or IFN score in association with systemic APS status were retrieved (**Supplementary Table S5**). However, the network analyses revealed noticeable differences in gene–gene interactions, as systemic APS patients showed a stronger and higher-degree network (**Figure 3A**). Nodes presenting with the higher correlations differed depending on systemic APS status (patients with and without systemic APS). The centrality measures supported these findings (**Figure 3B**), with IFI44, IFI44L, and MX1 showing the largest differences between groups, followed by OAS1 and RSAD2.

Finally, systemic APS related to a differential distribution of the clusters previously identified, being more likely to use clusters I and II, compared to those without systemic APS (*p* = 0.003) (**Figure 3C**). Importantly, the usage of these clusters was different from that of conventional APS. It is worth noting that when systemic APS diagnosis was added to the nosological/clinical groups, it segregated from PAPS and SAPS (**Supplementary Figure S1**), thereby confirming their differential status. Finally, it must be noted that these findings were obtained using a stringent definition of systemic APS. However, with a less strict definition (excluding ANA positivity), a slightly higher number of patients were classified as having systemic APS (*n* = 12), but equivalent findings for IFN-I pathway activation were obtained (data not shown).

These findings suggest that the systemic APS subset is hallmarked by a distinct IFN-I pathway activation profile, which can be attributed to a distinct coordinated expression of certain ISGs rather than their absolute expression values.

## 4 Discussion

The findings herein presented revealed overall a strong IFN-I pathway activation across the whole APS spectrum, even in individuals not fulfilling the classification criteria or clinical manifestations of APS but tested persistently positive for aPL. Moreover, a progressive activation increases from those individuals toward patients in whom a more complex clinical phenotype such as SAPS or SLE was observed, where the highest activation was registered. Although similar findings have been reported in isolated monocytes (17), these were not confirmed at the whole blood level. Importantly, monocytes represent only a fraction (and by no means the majority) of IFN-I-responding cells, so the actual relevance of these findings at the patient level is unknown, especially considering the effects of cytopenia or leukopenia in these patients. Furthermore, although some studies have addressed the analysis of IFN signatures or scores in PAPS and SAPS (18–20), evidence in aPL carriers is scarce and represents a major unmet need. The observation of an enhanced IFN-I pathway activation in those subjects only characterized by the presence of aPL without clinical manifestations represents an interesting tool for patients profiling and monitoring. Prospective studies are needed to demonstrate the usefulness of IFN-I pathway in predicting disease evolution and stratifying patients according to the risk of developing clinical manifestations, as reported in other scenarios (21–23). It is worth noting that a significant heterogeneity was observed within the aPL carriers subset in terms of genes and extent of activation, which may reflect its clinical within-group heterogeneity as it may be associated with different clinical trajectories. The findings from the cluster and correspondence analyses supported this idea.

Regarding individual ISGs trends, whereas some genes were increased in all subsets compared to HCs (such as IFI44L, MX1, and RSAD2), other genes were found to be increased only in SAPS and SLE patients (such as IFI6 or IFI27). These findings were paralleled, at least in part, by differences in influence in network analyses. Importantly, selected groups of ISGs (such as IFI44-OAS1) showed differential associations across subsets, hence suggesting preferential pathway trajectories. Taken together, these results may inform different expression programs specific for each disease subset. Although this notion had been hypothesized in previous studies (18), suboptimal reporting practices and little evidence have limited its appraisal. Our findings align with the idea that differential APS-specific components can be found within IFN-I fingerprints, probably in relation to distinct pathogenic substrates among related conditions (24, 25). Gaining understanding toward these trends will not only shed light into disease taxonomy but also provide a better understanding of the connections between clinically relevant signatures and nosological entities in APS and between APS and other systemic and rheumatic conditions hallmarked by IFN-I involvement.

A remarkable breakthrough from our study was the assessment of gene–gene correlations. The network analyses reinforced that different gene expression programs could be distinguished across the APS spectrum, which cannot be captured solely by analyzing the expression levels. Overall, our results unveil a significant heterogeneity among IFN-I pathway activation patterns within APS. This heterogeneity may be linked with different clinical values, hence explaining the diverging associations with clinical and serologic features, such as thrombotic events or autoantibody profiles, among APS subsets. A similar scenario has been reported in rheumatoid arthritis and SLE populations by our group (16, 26) and others (21, 27, 28), whereas this phenomenon had not been explored in APS to this date. This notion may explain the controversy observed in previous studies about the association between IFN-I pathway activation and clinical outcomes, such as the association with aβ2GPI antibodies (9, 29). We found no associations between IFN-I pathway activation and criteria aPL, either individually, as combined profiles (double or triple positivity), or as the number of antibodies. However, our study unveiled an association between IFN-I pathway activation and aPS/PT antibodies in both aPL carriers and PAPS patients. Taken together, these findings strengthen the connections between IFN-I signaling and humoral responses in APS and suggest that this association may be restricted to certain specificities rather than an overall unspecific effect. Previous evidence suggested that aPL can trigger IFNα production (30, 31). Whether this applies to aPS/PT antibodies requires further mechanistic research. On the other hand, these findings add to the emerging clinical relevance of the aPS/PT antibodies, as these may help to identify groups of patients with specific characteristics, including an elevated IFN-I pathway activation. While criteria aPL are still considered the mainstay for risk stratification, data supporting the additional role of scoring systems, such as the GAPSS and “extra-criteria” aPL, in specific subgroups of subjects, like those at high suspicion for APS diagnosis but tested negative for criteria aPL or when LA testing is not available, are rapidly growing (32–34). Finally, despite the lack of associations between IFN-I pathway activation and thrombotic events in our cohort, an increased expression of several ISGs (IFI44, OAS1, and RSAD2) and the IFN score was found in the SAPS subgroup in relation to recurrent thrombotic events. A more pronounced pro-coagulant and pro-inflammatory profile, derived from the association between SLE and APS, might at least partially explain the ability of the IFN-I pathway to capture the higher risk for thrombotic recurrences in this subset. However, further studies are warranted to clarify this observation.

Interestingly, our study also focused on the recently described systemic APS subset. Although proof-of-concept, our analyses revealed that patients considered as having systemic APS are hallmarked by specific gene–gene correlations and a differential usage of ISGs clusters, which segregate from those in APS (either aPL carriers, PAPS, or SAPS) and SLE populations, the involvement of IFN-I in this scenario aligns with the solid association of this mediator with the occurrence of autoantibodies and cytopenia in systemic conditions (35). Therefore, these data suggest that the IFN-I pathway may represent an innovative tool for identifying those patients who present with an intermediate clinical and serological phenotype between PAPS and SLE and who might benefit from other clinical management strategies. Although anti-aggregation and anticoagulation still represent the first therapeutic approaches in aPL-positive patients, our data could support the use of therapeutic and preventive approaches targeting the immune dysregulation, especially when systemic (clinical or laboratory) features are present. The employment of new treatment strategies directly or indirectly targeting the IFN-I pathway, including the use of anti-IFN-I antibodies (discussed below) or drugs with the ability to modulate the IFN-I activation, can be conceived based on our findings. Further prospective studies should be performed to translate this into clinical practice.

We must acknowledge that suggesting the use of IFN-I as a profiling tool while identifying an additional discrete subset, called systemic APS, might sound contradictory. Nevertheless, since the use of IFN-I pathway activation assays and molecular characterization for profiling purposes needs further investigation before overcoming the traditional categorization approach, classification criteria still represent a fundamental tool for practical clinical guidance and patients’ management. Taken together, our results further confirm the usefulness of IFN-I pathway activation in aPL-positive patient profiling, mirroring the role of this pathway in APS pathogenesis, and further demonstrate the existence of clinical phenotypes beyond traditional classification criteria.

Contemporary IFN research has been characterized by a large heterogeneity in terms of preclinical standardization, assay methodology, and clinical validation, which may account for its lack of translation into the clinical setting. Recently, EULAR recommendations to guide future steps on measurement, reporting, and application of IFN-I assays in clinical research and practice have been published (36). This study represents the first work uptaking these recommendations in APS, including a separate description of the IFN score, empirical support for composite score calculation, and uptake of consensus terminology. Moreover, the analysis of a less explored disease, such as APS, is compliant with the research agenda (7, 36).

Our study has certain limitations that should be acknowledged. First, the prevalence of obstetric manifestations in our cohort was low, thus limiting our ability to capture possible associations with pregnancy complications. In addition, the cross-sectional design of the study does not allow for the observation of variations and fluctuations in IFN-I pathway activation over time, therefore preventing a correlation with disease activity, clinical manifestations, and disease evolution. Finally, our cohort included a relatively low number of SLE patients under corticosteroid treatment, which may influence IFN-I pathway activation according to the literature. However, our results found no effect across groups in any of the ISGs analyzed, hence ruling out a major confounding effect in this context.

In conclusion, IFN-I pathway activation is a common hallmark across the APS spectrum, being found elevated even in those aPL-positive subjects who did not fulfil the classification criteria for the syndrome. Far from being a uniform expression program, different expression patterns could be distinguished, which may underlie the distinct clinical correlates among APS subsets. Finally, aPL-positive patients who present with a higher rate of systemic features, named systemic APS, exhibited a characteristic IFN-I pathway activation profile. To the best of our knowledge, this is the first study characterizing IFN-I pathway activation across the APS spectrum. These findings pave the ground for the translational use of IFN-I pathway activation in monitoring and risk profiling. Further larger and prospective studies are needed to evaluate the potential role of IFN-I pathway activation to predict thrombotic outcomes as well as disease evolution in aPL-positive patients. Furthermore, preclinical research has demonstrated beneficial effects of the abrogation of IFN-I signaling in APS (37), which adds to the successful results from phase III trials in SLE (38). Although anticoagulation stays as the therapeutic mainstay in APS, in the era of IFN-targeted therapies, it may be conceivable to evaluate the effects of IFN-I blockade in aPL-positive patients presenting with IFN-I pathway activation.

## Data availability statement

The raw data supporting the conclusions of this article will be made available by the authors without undue reservation.

## Ethics statement

The studies involving humans were approved by Institutional Review Boards from the University of Oviedo (reference CEImPA 2021.126). The studies were conducted in accordance with the local legislation and institutional requirements. The participants provided their written informed consent to participate in this study.

## Author contributions

IC: Writing – original draft, Investigation, Conceptualization. MR: Writing – review & editing, Investigation. AB: Writing – review & editing, Investigation. SF: Writing – review & editing, Resources. EM: Writing – review & editing. DR: Writing – review & editing, Supervision. AS: Writing – review & editing, Supervision. SS: Writing – review & editing, Conceptualization. JR-C: Writing – original draft, Supervision, Methodology, Formal Analysis.
