# Supplementary material for: Type I interferon pathway activation across the antiphospholipid syndrome spectrum: associations with disease subsets and systemic antiphospholipid syndrome presentation
Source: Front Immunol. 2024 Mar 14;15:1351446. doi: 10.3389/fimmu.2024.1351446 (PMC10972891; doi:10.3389/fimmu.2024.1351446)
Supplement: Supplementary file 1 [file DataSheet_1.docx]

**SUPPLEMENTARY MATERIAL**

**SUPPLEMENTARY TABLES**

**Supplementary Table S1: Associations between IFN-I pathway activation and thrombotic outcomes across APS subsets.** The associations between IFN-I pathway activation and thrombotic outcomes were evaluated by Mann-Withney U or Spearman’s rank tests, as appropriate. Associations reaching statistical significance were highlighted in bold. *IFN means interferon; APS, antiphospholipid syndrome; aPL+, antiphospholipid antibodies carriers; PAPS, primary APS; SAPS, secondary APS; SLE, systemic lupus erythematosus.*

|  | **IFI6** | **IFI44** | **IFI44L** | **MX1** | **IFI27** | **OAS1** | **RSAD2** | **IFN score** |
| --- | --- | --- | --- | --- | --- | --- | --- | --- |
| ***Arterial thrombosis*** | |  |  |  |  |  |  |  |
| aPL+ | p=0.593 | p=0.999 | p=0.963 | p=0.889 | p=0.593 | p=0.815 | p=0.999 | p=0.815 |
| PAPS | p=0.312 | p=0.258 | p=0.594 | p=0.921 | p=0.767 | p=0.567 | p=0.650 | p=0.489 |
| SAPS | p=0.880 | p=0.525 | p=0.449 | p=0.740 | p=0.211 | p=0.608 | p=0.608 | p=0.525 |
| SLE | p=0.750 | p=0.999 | p=0.667 | p=0.999 | p=0.250 | p=0.750 | p=0.999 | p=0.750 |
| ***Venous thrombosis*** | |  |  |  |  |  |  |  |
| aPL+ | p=0.091 | p=0.410 | p=0.365 | p=0.365 | p=0.462 | p=0.239 | p=0.462 | p=0.205 |
| PAPS | p=0.520 | p=0.830 | p=0.770 | p=0.861 | p=0.140 | p=0.953 | p=0.800 | p=0.626 |
| SAPS | p=0.534 | p=0.389 | p=0.376 | p=0.615 | p=0.501 | p=0.397 | p=0.640 | p=0.441 |
| SLE | p=0.145 | p=0.043 | p=0.087 | p=0.464 | p=0.217 | p=0.181 | p=0.145 | p=0.181 |
| ***Recurrences of thrombosis*** | | |  |  |  |  |  |  |
| PAPS | p=0.856 | p=0.999 | p=0.897 | p=0.448 | p=0.548 | p=0.696 | p=0.735 | p=0.938 |
| SAPS | p=0.067 | **p=0.037** | p=0.111 | p=0.080 | p=0.174 | **p=0.046** | p=0.067 | **p=0.050** |
| SLE | p=0.333 | p=0.083 | p=0.083 | p=0.583 | p=0.083 | p=0.250 | p=0.250 | p=0.167 |
| ***Number of recurrences of thrombosis*** | | |  |  |  |  |  |  |
| PAPS | r=-0.034  p=0.856 | r=0.003  p=0.987 | r=-0.021  p=0.911 | r=-0.137  p=0.462 | r=0.132  p=0.478 | r=-0.079  p=0.672 | r=-0.053  p=0.776 | r=-0.009  p=0.961 |
| SAPS | **r=0.397**  **p=0.050** | **r=0.445**  **p=0.033** | r=0.350  p=0.102 | r=0.381  p=0.053 | r=0.302  p=0.161 | **r=0.429**  **p=0.041** | **r=0.397**  **p=0.041** | **r=0.381**  **p=0.045** |
| SLE | r=0.256  p=0.227 | r=0.346  p=0.097 | r=0.346  p=0.097 | r=0.166  p=0.438 | r=0.347  p=0.097 | r=0.286  p=0.175 | r=0.286  p=0.175 | r=0.316  p=0.132 |

**Supplementary Table S2: Associations between IFN-I pathway activation and autoantibody profiles across APS subsets.** The associations between IFN-I pathway activation and autoantibody profiles were evaluated by Mann-Withney U or Spearman’s rank tests, as appropriate. Associations reaching statistical significance were highlighted in bold. *IFN means interferon; APS, antiphospholipid syndrome; aPL+, antiphospholipid antibodies carriers; PAPS, primary APS; SAPS, secondary APS; SLE, systemic lupus erythematosus; aCL, anti-cardiolipin antibodies; LA, lupus anticoagulant; aβ2GPI, anti-β2 glycoprotein I; ANA, anti-nuclear antibodies; aPS/PT, anti-phosphatidylserine/prothrombin antibodies.*

|  | **IFI6** | **IFI44** | **IFI44L** | **MX1** | **IFI27** | **OAS1** | **RSAD2** | **IFN score** |
| --- | --- | --- | --- | --- | --- | --- | --- | --- |
| ***aCL*** | |  |  |  |  |  |  |  |
| aPL+ | p=0.458 | p=0.999 | p=0.999 | p=0.905 | p=0.756 | p=0.867 | p=0.583 | p=0.943 |
| PAPS | p=0.317 | p=0.444 | p=0.253 | p=0.274 | p=0.999 | p=0.417 | p=0.274 | p=0.341 |
| SAPS | p=0.083 | p=0.250 | p=0.201 | p=0.734 | p=0.224 | p=0.250 | p=0.688 | p=0.250 |
| ***LA*** | |  |  |  |  |  |  |  |
| aPL+ | p=0.130 | p=0.685 | p=0.198 | p=0.198 | p=0.145 | p=0.198 | p=0.288 | p=0.240 |
| PAPS | p=0.502 | p=0.764 | p=0.764 | p=0.945 | p=0.999 | p=0.182 | p=0.872 | p=0.945 |
| SAPS | **p=0.012** | p=0.054 | **p=0.035** | **p=0.009** | p=0.324 | p=0.218 | **p=0.021** | p=0.067 |
| ***αβ2GPI*** | | |  |  |  |  |  |  |
| aPL+ | p=0.223 | p=0.141 | p=0.359 | p=0.359 | p=0.359 | p=0.537 | p=0.604 | p=0.309 |
| PAPS | p=0.370 | p=0.663 | p=0.546 | p=0.519 | p=0.053 | p=0.999 | p=0.787 | p=0.441 |
| SAPS | p=0.235 | p=0.211 | p=0.169 | p=0.740 | p=0.118 | p=0.190 | p=0.413 | p=0.169 |
| ***ANA*** |  |  |  |  |  |  |  |  |
| aPL+ | **p=0.002** | **p=0.013** | **p=0.009** | **p=0.023** | **p<0.001** | **p=0.001** | **p=0.011** | **p=0.004** |
| PAPS | p=0.151 | p=0.247 | p=0.247 | p=0.202 | p=0.572 | p=0.446 | p=0.495 | p=0.188 |
| SAPS | p=0.273 | p=0.727 | p=0.727 | p=0.727 | p=0.727 | p=0.545 | p=0.545 | p=0.636 |
| ***aPS/PT IgM*** | | |  |  |  |  |  |  |
| aPL+ | r=0.379  p=0.075 | r=0.369  p=0.083 | **r=0.422**  **p=0.045** | **r=0.434**  **p=0.045** | r=0.272  p=0.209 | r=0.302  p=0.209 | r=0.302  p=0.161 | r=0.389  p=0.067 |
| PAPS | r=0.173  p=0.387 | r=0.218  p=0.275 | r=0.228  p=0.252 | r=0.189  p=0.345 | r=0.332  p=0.091 | r=-0.116  p=0.563 | r=0.180  p=0.370 | r=0.180  p=0.370 |
| SAPS | r=0.230  p=0.374 | r=0.113  p=0.667 | r=0.088  p=0.736 | r=0.228  p=0.379 | r=0.306  p=0.232 | r=0.078  p=0.765 | r=0.147  p=0.573 | r=0.157  p=0.548 |
| SLE | r=0.020  p=0.929 | r=0.156  p=0.477 | r=0.171  p=0.435 | r=0.174  p=0.427 | r=0.278  p=0.200 | r=0.189  p=0.388 | r=0.194  p=0.376 | r=0.186  p=0.396 |
| ***aPS/PT IgG*** | |  |  |  |  |  |  |  |
| aPL+ | **r=0.571**  **p=0.006** | **r=0.621**  **p=0.002** | **r=0.585**  **p=0.004** | **r=0.586**  **p=0.004** | r=0.150  p=0.506 | **r=0.591**  **p=0.004** | **r=0.576**  **p=0.005** | **r=0.606**  **p=0.003** |
| PAPS | **r=0.404**  **p=0.037** | **r=0.390**  **p=0.044** | r=0.310  p=0.116 | r=0.241  p=0.226 | r=0.372  p=0.056 | r=-0.010  p=0.959 | r=0.266  p=0.180 | r=0.295  p=0.135 |
| SAPS | r=-0.038  p=0.880 | r=0.119  p=0.639 | r=0.024  p=0.926 | r=0.185  p=0.463 | r=-0.232  p=0.354 | r=0.220  p=0.381 | r=0.168  p=0.505 | r=0.063  p=0.804 |
| SLE | r=-0.069  p=0.755 | r=0.001  p=0.998 | r=0.126  p=0.567 | r=0.037  p=0.868 | r=-0.085  p=0.701 | r=0.094  p=0.668 | r=0.121  p=0.582 | r=0.059  p=0.788 |

**Supplementary Table S3: Associations between IFN-I pathway activation and combined autoantibody profiles across APS subsets.** The associations between IFN-I pathway activation and combined autoantibody profiles were evaluated by Mann-Withney U or Spearman’s rank tests (p for trend), as appropriate. Double positivity was defined at having at least two criteria aPL. Triple positivity was defined as having three criteria aPL. Numbers of patients exhibiting these combinations are indicated for each APS subset. Associations reaching statistical significance were highlighted in bold. *IFN means interferon; APS, antiphospholipid syndrome; aPL+, antiphospholipid antibodies carriers; PAPS, primary APS; SAPS, secondary APS; SLE, systemic lupus erythematosus.*

|  | **IFI6** | **IFI44** | **IFI44L** | **MX1** | **IFI27** | **OAS1** | **RSAD2** | **IFN score** |
| --- | --- | --- | --- | --- | --- | --- | --- | --- |
| ***Double positivity (yes vs no)*** | |  |  |  |  |  |  |  |
| aPL+ (14 vs 15) | p=0.217 | p=0.614 | p=0.648 | p=0.648 | p=0.427 | p=0.648 | p=0.905 | p=0.548 |
| PAPS (23 vs 8) | p=0.437 | p=0.642 | p=0.808 | p=0.707 | p=0.317 | p=0.774 | p=0.877 | p=0.642 |
| SAPS (14 vs 11) | p=0.403 | p=0.403 | p=0.369 | p=0.926 | p=0.250 | p=0.305 | p=0.781 | p=0.305 |
| ***Triple positivity (yes vs no)*** | |  |  |  |  |  |  |  |
| aPL+ (5 vs 24) | **p=0.033** | **p=0.019** | **p=0.023** | **p=0.028** | p=0.257 | p=0.113 | p=0.086 | **p=0.050** |
| PAPS (15 vs 16) | p=0.922 | p=0.711 | p=0.740 | p=0.470 | p=0.338 | p=0.129 | p=0.520 | p=0.572 |
| SAPS (10 vs 15) | p=0.257 | p=0.186 | p=0.148 | p=0.999 | p=0.075 | p=0.166 | p=0.605 | p=0.232 |
| ***Number of autoantibodies*** | |  |  |  |  |  |  |  |
| aPL+ | r=0.357  p=0.075 | r=0.207  p=0.301 | r=0.226  p=0.256 | r=0.233  p=0.242 | r=0.207  p=0.301 | r=0.205  p=0.304 | r=0.093  p=0.643 | r=0.232  p=0.244 |
| PAPS | r=0.048  p=0.798 | r=-0.012  p=0.950 | r=-0.024  p=0.897 | r=-0.067  p=0.733 | r=0.206  p=0.267 | r=-0.220  p=0.234 | r=-0.071  p=0.703 | r=-0.037  p=0.844 |
| SAPS | r=-0.150  p=0.496 | r=-0.148  p=0.500 | r=-0.164  p=0.455 | r=0.090  p=0.682 | r=-0.257  p=0.236 | r=-0.196  p=0.369 | r=0.004  p=0.985 | r=-0.160  p=0.465 |

**Supplementary Table S4: Associations between IFN-I pathway activation and treatments across APS subsets.** The associations between IFN-I pathway activation and treatments were evaluated by Mann-Withney U tests. Associations reaching statistical significance were highlighted in bold. *IFN means interferon; APS, antiphospholipid syndrome.*

|  | **IFI6** | **IFI44** | **IFI44L** | **MX1** | **IFI27** | **OAS1** | **RSAD2** | **IFN score** |
| --- | --- | --- | --- | --- | --- | --- | --- | --- |
| ***Prednisone or equivalent ≤ 5 mg/day*** | |  |  |  |  |  |  |  |
| aPL+ | p=0.495 | p=0.743 | p=0.561 | p=0.781 | p=0.232 | p=0.433 | p=0.403 | p=0.463 |
| PAPS | p=0.827 | p=0.789 | p=0.865 | p=0.906 | p=0.999 | p=0.715 | p=0.751 | p=0.981 |
| SAPS | p=0.833 | p=0.928 | p=0.928 | p=0.651 | p=0.880 | p=0.928 | p=0.880 | p=0.928 |
| SLE | p=0.516 | p=0.431 | p=0.227 | p=0.473 | p=0.759 | p=0.256 | p=0.354 | p=0.392 |
| ***HCQ (200-400 mg/day)*** | | |  |  |  |  |  |  |
| aPL+ | p=0.232 | p=0.668 | p=0.495 | p=0.348 | p=0.322 | p=0.275 | p=0.561 | p=0.375 |
| PAPS | p=0.729 | p=0.695 | p=0.999 | p=0.945 | p=0.595 | p=0.999 | p=0.800 | p=0.945 |
| SAPS | p=0.769 | p=0.579 | p=0.452 | p=0.376 | p=0.413 | p=0.413 | p=0.278 | p=0.341 |
| SLE | p=0.608 | p=0.347 | p=0.566 | p=0.316 | p=0.413 | p=0.413 | p=0.379 | p=0.487 |
| ***LDA (100 mg/day)*** | | |  |  |  |  |  |  |
| aPL+ | p=0.202 | p=0.616 | p=0.458 | p=0.867 | p=0.094 | p=0.325 | p=0.519 | p=0.402 |
| PAPS | p=0.244 | p=0.157 | p=0.227 | p=0.381 | p=0.338 | p=0.197 | p=0.261 | p=0.227 |
| SAPS | p=0.999 | p=0.877 | p=0.926 | p=0.734 | p=0.829 | p=0.877 | p=0.781 | p=0.877 |
| SLE | p=0.505 | p=0.907 | p=0.725 | p=0.845 | p=0.725 | p=0.999 | p=0.907 | p=0.725 |
| ***Vitamin K antagonists*** | |  |  |  |  |  |  |  |
| aPL+ | - | - | - | - | - | - | - | - |
| PAPS | p=0.826 | p=0.617 | p=0.795 | p=0.459 | p=0.704 | p=0.287 | p=0.589 | p=0.734 |
| SAPS | p=0.190 | p=0.288 | p=0.413 | p=0.316 | p=0.566 | p=0.211 | p=0.288 | p=0.413 |
| SLE | - | - | - | - | - | - | - | - |
| ***Other immunosuppressive treatment*** | | |  |  |  |  |  |  |
| aPL+ | - | - | - | - | - | - | - | - |
| PAPS | - | - | - | - | - | - | - | - |
| SAPS | p=0.140 | p=0.161 | p=0.161 | p=0.121 | p=0.860 | p=0.140 | p=0.104 | p=0.140 |
| SLE | p=0.060 | p=0.070 | p=0.148 | p=0.057 | p=0.131 | p=0.131 | p=0.148 | p=0.098 |

**Supplementary Table S5: IFN pathway activation in systemic APS.** The expression of individual ISGs (Z-scores) and the composite IFN score were evaluated according to systemic APS status by Mann-Withney U tests. Variables are summarized as median (Interquartile range). Associations reaching statistical significance were highlighted in bold. *IFN means interferon; APS, antiphospholipid syndrome.*

|  | **Systemic APS** | | **p-value** |
| --- | --- | --- | --- |
|  | **No (n=48)** | **Yes (n=9)** |  |
| **IFI6** | -0.57 (0.45) | -0.21 (0.46) | 0.185 |
| **IFI44** | -0.50 (0.15) | -0.39 (0.33) | 0.292 |
| **IFI44L** | -0.48 (0.11) | -0.42 (0.20) | 0.397 |
| **MX1** | -0.55 (0.49) | -0.39 (0.58) | 0.261 |
| **IFI27** | -0.37 (0.03) | -0.36 (0.04) | 0.129 |
| **OAS1** | -0.48 (0.58) | -0.32 (0.35) | 0.948 |
| **RSAD2** | -0.49 (0.20) | -0.39 (0.22) | 0.515 |
| **IFN score** | -0.48 (0.24) | -0.37 (0.27) | 0.328 |

**SUPPLEMENTARY FIGURES**

**Supplementary Figure 1: Correspondence analysis of systemic APS and APS subsets.** The usage of the three clusters (black dots) depending of systemic APS status was evaluated by correspondence analysis. Patients presenting with systemic APS are grouped as systemic APS (orange square), whereas those without are grouped according to the conventional classification (PAPS, SAPS, SLE and HCs) (gray squares). *APS means antiphospholipid syndrome; PAPS, primary APS; SAPS, secondary APS; SLE, systemic lupus erythematosus; HCs, healthy controls.*
