# Supplementary figures and images for: Type I interferon pathway activation across the antiphospholipid syndrome spectrum: associations with disease subsets and systemic antiphospholipid syndrome presentation
Source: Front Immunol. 2024 Mar 14;15:1351446. doi: 10.3389/fimmu.2024.1351446 (PMC10972891; doi:10.3389/fimmu.2024.1351446)

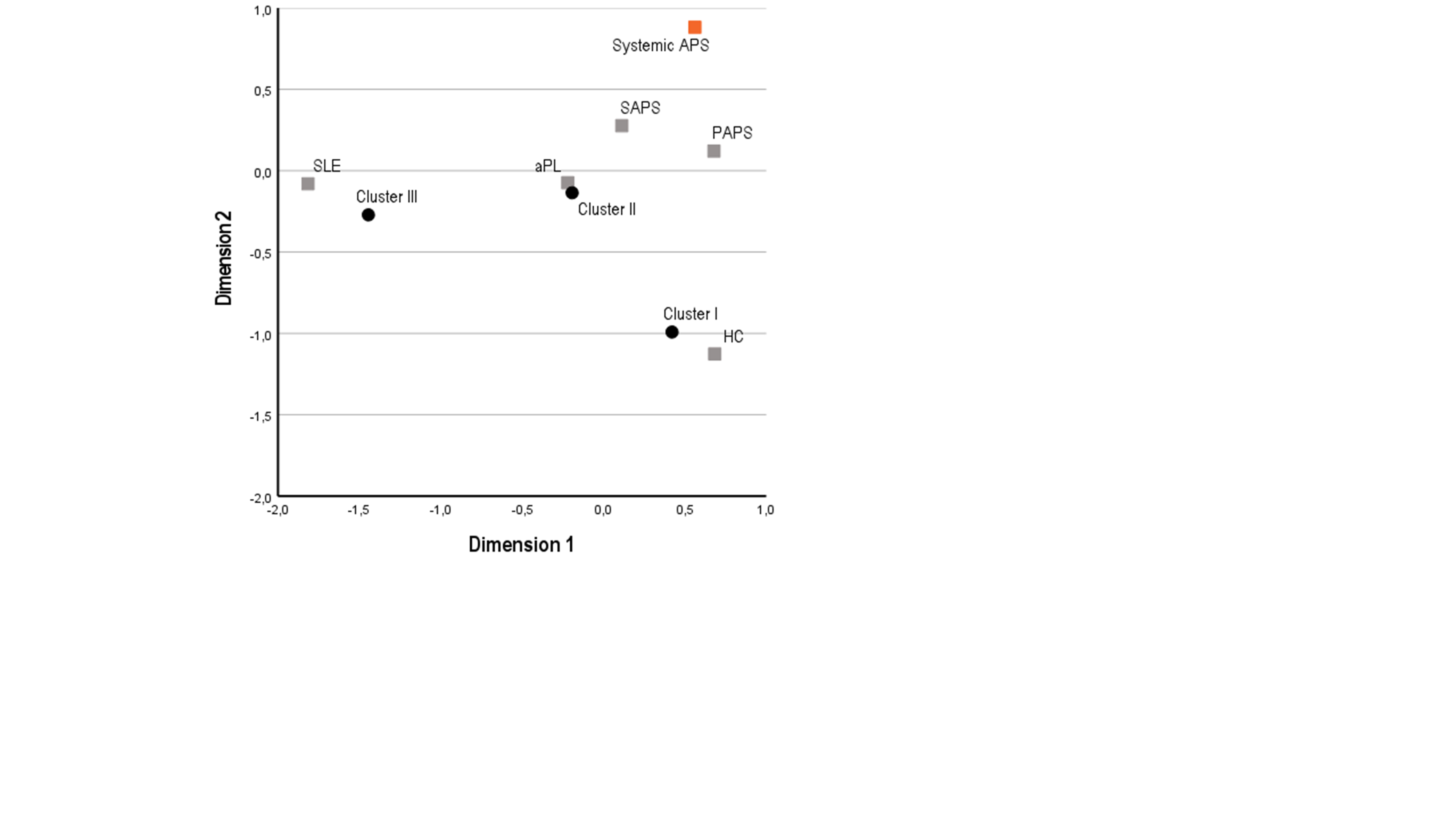

Supplement: Supplementary file 2 [file Image_1.tif]
